# Converter Phenotype: A New Profile That Is Not Exclusive to Taxanes

**DOI:** 10.3389/falgy.2021.785259

**Published:** 2022-01-12

**Authors:** Teodorikez Wilfox Jimenez-Rodriguez, Francisco Manuel Marco de la Calle, Inmaculada Lozano-Cubo, Rosa Ana Montoyo-Anton, Victor Soriano-Gomis, Purificación Gonzalez-Delgado, Amparo Burgos-San José, Seira Climent-Ballester, Natividad Martínez-Banaclocha, Javier Fernández-Sanchez

**Affiliations:** ^1^Allergy Section, Alicante General University Hospital, Alicante Institute for Health and Biomedical Research (ISABIAL), UMH, Alicante, Spain; ^2^Spanish Research Network on Asthma and Adverse and Allergic Reactions (ARADyAL) Spanish Network (RD16/0006), Instituto de Salud Carlos III, Fundación Española para la Ciencia y la Tecnología, Madrid, Spain; ^3^Immunology Service, Alicante General University Hospital, Alicante Institute for Health and Biomedical Research (ISABIAL), Alicante, Spain; ^4^Oncology Section, Alicante General University Hospital, Alicante Institute for Health and Biomedical Research (ISABIAL), Alicante, Spain; ^5^Oncology Day Hospital Nursing Service, Alicante General University Hospital, Alicante Institute for Health and Biomedical Research (ISABIAL), Alicante, Spain; ^6^Pharmacy Department, Alicante General University Hospital, Alicante Institute for Health and Biomedical Research (ISABIAL), Alicante, Spain

**Keywords:** rapid drug desensitization, hypersensitivity, oncoimmunology, chemotherapy (CH), monoclonal antibodies (immunology), desensitization and challenge, drug allergy, drug hypersensitivity reaction (DHR)

## Abstract

**Introduction:** Phenotype I hypersensitivity reactions are the most commonly reported drug reactions; however, precision medicine has made it possible to characterize new phenotypes. A recent communication proposed the existence of a “converter phenotype,” which would affect patients who present non-immediate hypersensitivity reactions and in subsequent exposures develop immediate hypersensitivity reactions. This study aimed to describe the clinical characteristics of converter phenotype reactions and their evolution during desensitization to chemotherapeutic drugs and monoclonal antibodies.

**Methods:** We retrospectively reviewed our database of patients undergoing desensitization to chemotherapy or biological agents and selected those with a converter phenotype. Demographic and clinical characteristics of the patients, the results of skin tests, tryptase and IL-6 levels, and desensitization outcomes were assessed.

**Results:** Of 116 patients evaluated, 12 (10.3%) were identified as having a converter phenotype. The median interval between drug exposure and reaction was 90.6 h (range 8-288 h). After the conversion, phenotype I was the most frequent (58.3%), followed by cytokine release reactions (33.3%). Fifty-one desensitizations were undertaken and all treatments completed, with 10 (19.6%) breakthrough reactions. No new changes in the phenotype were detected.

**Conclusions:** The symptoms of non-immediate drug hypersensitivity reactions may indicate the need for an early allergological evaluation to assess the risk of future immediate drug reactions. Clinical characteristics, skin test results, and biomarkers can help predict responses to rapid drug desensitization, guiding clinicians on how to optimize therapy delivery while maintaining patient safety.

## Introduction

Phenotype I (PhI) reactions are the most commonly reported kinds of drug hypersensitivity reactions (DHR) according to the Gell and Coombs' classic description; however, precision medicine has brought to light other types that had no place into this classification, such as cytokine release reactions (1). Gell and Coombs' classification also left out some phenotypes of non-immediate drug hypersensitivity reactions (NIDHR), including accelerated reactions (2, 3).

NIDHR occur any time as from 1 h after the initial drug administration, frequently appearing after many days of treatment (4), and include both accelerated reactions that appear 1-48 h after exposure to the drug and delayed reactions which occur after 72 h (2, 3). Although NIDHRs are less frequent than their immediate counterparts (IDHRs), they represent a diagnostic and therapeutic challenge because few patients who develop this group of reactions can continue with their first line of treatment (5).

In 2018, preliminary clinical descriptions about a new phenotype of DHRs to taxanes emerged (6). The so-called the “converter phenotype” (CPh) defined a group of taxane-treated patients who initially presented with NIDHRs but developed IDHRs after subsequent exposures, generally PhI reactions (6).

Previous publications have demonstrated the benefits of rapid drug desensitization for treating several phenotypes, including PhI, cytokine release reactions (CRRs), mixed phenotypes (MxPh) and NIDHRs (1, 7–11). Nevertheless, desensitization is contraindicated in Gell and Coombs' type II DHRs, type III DHRs, and severe cutaneous adverse reactions (SCARs), which are a subtype of type IV DHRs (5, 12).

The pathophysiological mechanisms that induce allergen-specific and temporal immunotolerance in PhI reactions include the inhibition of both the early and late response of the mast cells (7, 13–15). Initially it was thought that it was because the internalization of antigen/IgE/FcεRI complexes (7, 13, 16), but recent studies suggest that the main mechanisms include the decrease in some signal transducer molecules such as Syk (17, 18), and the recruitment of the inositol phosphatase SHIP-1 into the plasma membrane, which could tip the balance between positive and negative signaling pathways that regulate degranulation (19).

On the other hand, the mechanisms for achieving tolerance during desensitizations in NIDHRs are poorly understood; previous studies have pointed to the activation of T lymphocytes as the main endotype (20, 21). In fact, one study of fixed drug eruptions suggest that the migration of CD25+ CD4+ T lymphocytes to the lesion during desensitization would modulate the response by suppressing the effector function of the CD8+ T lymphocytes that are responsible for the initial lesion (22).

Recent analysis of cytokines during both IDHR and NIDHR shows substantial increases in IL-10 after desensitization, related to the action of regulatory T lymphocytes and regulatory B lymphocytes, meaning that tolerance induced by desensitization implies the modulation of drug-specific response by regulatory mechanisms (23).

Little is known about the mechanisms involved in CPh reactions. However, a previous series of cases presented in poster form on the European Academy of Allergy and Clinical Immunology Annual Congress, suggested that desensitization could be beneficial in these patients, potentially affecting the switch from NIDHR to IDHR (6).

This study aimed to describe the clinical characteristics of CPh reactions and their evolution during desensitization, not only with taxanes but also with other chemotherapeutic drugs and monoclonal antibodies (MAb) that have not yet been associated with this phenotype in the literature.

## Methods

We retrospectively analyzed the medical records of all patients who underwent desensitization to chemotherapeutic or biological drugs at the Alicante General University Hospital between January 2019 and December 2020. Only patients who presented symptoms compatible with CPh were included in the analysis. Patients with non-immediate, severe skin reactions with systemic symptoms (SCARS) were excluded. The Hospital Ethics Committee approved the study protocol.

We considered a CPh in patients who experienced an IDHR to chemotherapy or MAbs after having experienced a NIDHR on the previous administration of that same drug (6). DHRs were classified as immediate when symptoms appeared 1 h to 6 h after exposure, or as non-immediate when they appeared at least 6 h after exposure (8). Previous studies classified IDHRs into phenotypes PhI, CRR, MxPh, or either phenotype (EPh) based on clinical symptoms (24, 25). Characteristic Type I symptoms include: pruritus, urticaria, angioedema, nasal congestion, sneezing, wheezing, cough, throat tightness, and tongue swelling—all related to the mast cell/basophil activation endotype. Symptoms of CRRs include chest pain, back pain, headache, rigors, other pain, chills, and fever; these are related to the monocytes, macrophages or T lymphocyte activation endotype. In addition, some non-specific symptoms are associated with both type I reactions and CRRs, making it impossible to differentiate the probable endotypes of the reactions; these include flushing/warmth/erythema, rash, dyspnea, oxygen desaturation, chest tightness, tachycardia, presyncope, syncope, hypertension, hypotension, nausea/vomiting, diarrhea, abdominal pain, bleeding, reflux, numbness/weakness, seizures, unusual taste, and diaphoresis.

Thus, the PhI phenotype involves type I reactions, with or without non-specific symptoms; the CRR phenotype implies CRR symptoms, with or without unspecific symptoms; the MxPh involves any combination of type I and/or CRR symptoms, with or without unspecific symptoms; and EPh involves only non-specific symptoms.

Patient age, sex, diagnosis, history of atopy, previous lifetime exposures to the culprit drug before the initial delayed reaction, interval of the delayed reaction, delayed symptoms, exposures to the culprit drug at the moment of the immediate reaction, grade of the immediate reaction classified according to Brown's grading system (26), results of skin tests (ST), tryptase and IL-6 levels, additional changes in the phenotype during the breakthrough reaction (BTR), and desensitization outcomes were assessed.

Atopy was defined as a confirmed history of at least one of the following: allergic rhinitis/asthma, food allergies, contact dermatitis, acute urticaria with an allergic cause, or allergy to Hymenoptera venom. Confirmed and unconfirmed allergies to other drugs were also evaluated.

Skin tests were performed at least 2 weeks after the last DHR, using, at prick level: paclitaxel in concentration of 6 mg/ml, docetaxel 10 mg/ml, carboplatin 10 mg/ml, oxaliplatin 5 mg/ml, rituximab 10 mg/ml, adalimumab 50 mg/ml, and brentuximab 5 mg/ml. If the prick was negative, dilutions of 1:1,000, 1:100, 1:10, or 1:1 were used for intradermal tests, as described elsewhere (25, 27). A wheal with a diameter at least 3 mm larger than that produced by a negative control (normal saline) and surrounded by erythema within 20 min of application was considered a positive result. Histamine skin prick testing (10 mg/ml) was used as a positive control. In addition, a reading took place at 24 and 48 h after skin testing. In all cases, we advised patients to monitor the delayed response for up to 1 week after testing.

The desensitization protocols used were those proposed by the Brigham and Women's Hospital group, consisting of bags, at different dilutions, administered in 4, 8, 12, or 16 steps with 2- to 2.5-fold dose increments, along with increasing infusion rate every 15 min, except in the final step, where it was maintained until the full dose was reached (7).

Based on the symptoms during the IDHRs, premedication was administered 30 min before desensitization, including cetirizine (10 mg orally) and ranitidine (50 mg intravenously). Additional premedication included aspirin 300 mg (for flushing) and montelukast 10 mg (for bronchospasm/chest tightness). COX-1 inhibitors, acetaminophen, fluids, and opioids were used to prevent chills, rigors, fever, and pain, while benzodiazepines (alprazolam 0.5 mg) were administered in case of anxiety. Patients were asked to withhold β-adrenergic blocking medications for 24 h before desensitization (10).

If a BTR occurred during desensitization, the infusion was stopped, and medications were administered based on symptoms and severity. Once the reaction resolved, the infusion was resumed at the step where it was stopped. Tryptase and IL-6 were drawn 30-120 min postreaction.

Serum tryptase and IL-6 levels were available for some patients at baseline and during the first IDHR; however, we were unable to obtain biomarkers for all reactions because other attending health professionals did not always order them, and in some cases there were patient care and time limitations.

Tryptase and IL-6 were determined using commercially available immunoassays following the manufacturer's instructions (Tryptase fluoroimmunoassay, Thermo Fisher Scientific, Uppsala, Sweden; and Elecsys IL-6 Immunoassay, Roche Diagnostics, USA, respectively). A serum tryptase concentration > 11.4 μg/L was considered elevated, as were the values during the reaction which exceeded the product obtained by the formula *Basal Tryptase x 1.2* + *2* (28, 29). IL-6 values more than 10 pg/ml were also considered elevated.

Descriptive statistics of categorical variables were expressed through absolute and relative counts using frequency tables and graphs. Quantitative variables were described using measures of central tendency (mean and median) and dispersion [standard deviation (SD) and interquartile range (IQR) and/or range]. We used the IBM SPSS Statistics program, version 27 (IBM Corp., Armonk, N.Y., USA).

## Results

Of the 116 patients evaluated for DHR to chemotherapeutic and biological drugs, 12 (10.3%) met criteria for CPh: 3 (25%) had reactions to paclitaxel, 3 (25%) to docetaxel, 2 (16.7%) to rituximab, 1 (8.3%) to adalimumab, 1 (8.3%) to brentuximab, 1 (8.3%) to carboplatin, and 1 (8.3%) to oxaliplatin.

Most (91.7%) of the included patients were women, and the mean age at diagnosis was 41 (SD 13.6) years. Ten (83.3%) patients were diagnosed with cancer (3 ovarian, 3 breast, 2 lymphomas, 1 endometrial cancer, and 1 gastric cancer), and two had autoimmune diseases (Crohn's disease and multiple sclerosis). Atopy was present in 80% of the patients.

The median number of drug exposures at the time of the NIDHR was 2.1 (range 0-16), and the median interval between drug exposure and reaction was 90.6 h (range 8-288 h). Patients reacting to taxanes had the least historical exposure to these drugs (83.3% had a reaction on their first exposure), while the carboplatin-allergic patient had been the most exposed (16 previous exposures prior to DHR). Table 1 summarizes the characteristics of the patients and the NIDHRs.

**Table 1:** Characteristics of included patients and non-immediate drug hypersensitivity reactions.

**Case**	**Sex**	**Age (years)**	**Ethnicity**	**Diagnosis**	**Stage**	**Type**	**Culprit drug**	***N* exposures before delayed reaction**	**Reaction interval (hours)**	**Delayed symptoms**	**Atopy**
1	F	39	White	Crohn's disease	NA	NA	Adalimumab	6	11	Erythema and swelling at injection site	Allergic rhinitis, drug allergy, urticaria
2	F	41	White	Ovarian cancer	III	Primary	Paclitaxel	0	120	Maculopapular exanthema	Asthma
3	F	40	White	Ovarian cancer	III	Primary	Paclitaxel	1	13	Maculopapular exanthema	Drug allergy
4	F	47	White	Breast cancer	II	Primary	Docetaxel	0	8	Flushing	Allergic rhinitis, drug allergy
5	F	48	White	Ovarian cancer	IV	Primary	Paclitaxel	0	72	Maculopapular exanthema	Allergic rhinitis, drug allergy
6	F	25	White	Multiple sclerosis	NA	NA	Rituximab	0	168	Malaise, myalgia, arthralgia	Allergic rhinitis, urticaria
7	F	18	Hispanic	Hodgkin lymphoma	IV	Recurrent	Brentuximab	0	48	Fever, chest pain, dyspnea, maculopapular exanthema	—
8	F	50	White	Breast cancer	II	Primary	Docetaxel	0	72	Maculopapular exanthema, abdominal pain, chest tightness	Allergic rhinitis
9	F	64	White	Endometrial cancer	IV	Recurrent	Carboplatin	16	48	Flushing, pruritus	Allergic rhinitis, drug allergy
10	F	41	White	Breast cancer	NA	Primary	Docetaxel	0	120	Chest pain	Allergic rhinitis
11	F	25	Hispanic	Gastric cancer	IV	Primary	Oxaliplatin	2^*^	120	Palmoplantar erythema, scaling	—
12	M	58	White	B-cell non-Hodgkin lymphoma	II	Primary	Rituximab	0	288	Fever, maculopapular exanthema, malaise, myalgia, arthralgia	—

*F, female; M, male; NA, not applicable/available*.

* *This patient tolerated 2 doses without problems, however from the 3th dose to the 6th dose developed with each exposure a NIDHR with the characteristics described*.

At the time of conversion, the mean exposure before developing the IDHR was 3.8 (range 1-17) and the phenotypes expressed during the IDHR were PhI (*n* = 7; 58.3%), CRR (*n* = 4; 33.3%) and EPh (*n* = 1; 8.3%).

The seven patients expressing PhI had an average of five prior lifetime exposures, most of which were grade (G) 2 (57.1%) or G3 (28.6%); the remaining 14.3% had a G1 reaction. Skin tests were positive in 6 (85.7%) patients. Reactions were mainly (57.1%) triggered by biological drugs (2 rituximab, 1 adalimumab, 1 brentuximab), followed by platins (28.6%: 1 carboplatin and 1 oxaliplatin) and taxanes (14.3%, 1 docetaxel) (Table 2).

**Table 2:** Characteristics of included patients at the moment of conversion to immediate hypersensitivity reactions.

**Case**	**Baseline tryptase (μg/L)**	**Baseline IL-6 (pg/mL)**	**Culprit drug**	**N exposures before immediate reaction**	**Immediate symptoms**	**Phenotype**	**Grade severity**	**Tryptase during IDHR (μg/L)**	**IL-6 during IDHR (pg/mL)**	**Skin test**
1	4.9		Adalimumab	7	Angioedema, pruritus, erythema	PhI	2			+
2	6.2		Paclitaxel	1	Flushing, back pain, chest tightness, abdominal pain	CRR	2			–
3	4.9		Paclitaxel	2	Flushing, back pain	CRR	2			–
4	4.5		Docetaxel	1	Flushing, pruritus, dyspnea, chest tightness, oxygen desaturation, abdominal pain, tremors, dizziness	PhI	3			+
5	2.6		Paclitaxel	1	Warmth, chest tightness, hypertension	EPh	2			–
6	3.9	12	Rituximab	1	Urticaria, pruritus, abdominal pain	PhI	2			+
7	4.4	8	Brentuximab	1	Pruritus, dyspnea, chest tightness, hypotension, oxygen desaturation	PhI	3	6.2	10	+
8	3	<2	Docetaxel	1	Flushing, fever	CRR	2	4.2	78	–
9	5.6	9	Carboplatin	17	Pruritus, flushing, chest tightness, dyspnea, tachycardia, blurred vision, diaphoresis, fainting	PhI	2			+
10			Docetaxel	1	Back pain, chest pain, chest tightness, dyspnea, dizziness, fainting	CRR	2			–
11	3	4	Oxaliplatin	7	Burning feeling, pruritus, sialorrhea, nasal blockage, cough, dyspnea, nausea, vomiting, blurred vision	PhI	2			+
12			Rituximab	1	Urticaria, angioedema, pruritus	PhI	1			–

*IL-6, interleukin 6; IDHR, Immediate drug hypersensitivity reaction; PhI, phenotype I; CRR, cytokine release reactions; EPh, either phenotype*.

On the other hand, patients who developed CRRs had an average of 1.3 exposures to the drug at the time of the IDHR, all corresponding to taxanes (50% to paclitaxel and 50% to docetaxel), with G2 reactions and negative STs.

Finally, one patient with an EPh developed a G2 reaction during the second exposure to paclitaxel, with a negative skin test. Clinical characteristics are summarized in Table 2.

Regardless of phenotype, none of the patients had positive results on the delayed reading of the skin tests.

Altogether, 51 desensitizations were done, and all (100%) of the treatments were completed. There were 10 (19.6%) BTRs: 7 (70%) of which were immediate and 3 (30%) non-immediate.

Regarding the phenotype expressed during the immediate BTRs, 5 (85.7%) were PhI, and 1 (14.3%) Eph; all were of the same phenotype as the IDHRs. On the other hand, non-immediate BTRs came from CRR in two patients who developed warmth and back pain some hours after desensitization. One carboplatin-allergic patient (patient 9 in all tables), developed a non-immediate BTR with fever and back pain a few hours after desensitization was completed; this patient developed an immediate PhI reaction during desensitization (Table 3). There were no immediate life-threatening or severe NIDHRs during or after desensitization.

**Table 3:** Characteristics of the desensitizations.

					**Characteristics of breakthrough reactions**						
**Case**	**Culprit drug**	**Initial grade severity**	**Initial phenotype**	***N* desensitizations**	***N* immediate reactions**	**Immediate symptoms**	**Phenotype**	**Tryptase** **(μg/L)**	**IL-6 (pg/ml)**	***N* non-immediate reactions**	**Non-immediate symptoms**
1	Adalimumab	2	PhI	1	0	—	—	—	—	0	—
2	Paclitaxel	2	CRR	4	0	—	—	—	—	1	Back pain
3	Paclitaxel	2	CRR	3	0	—	—	—	—	0	—
4	Docetaxel	3	PhI	1	0	—	—	—	—	0	—
5	Paclitaxel	2	EPh	6	1	Chest tightness	EPh	NA	NA	0	—
6	Rituximab	2	PhI	1	1	Urticaria, pruritus, feeling of pharyngeal edema, dysphagia	PhI	3.1	14	0	—
7	Brentuximab	3	PhI	15	0	—				0	—
8	Docetaxel	2	CRR	4	0	—				1	Warmth, back pain
9	Carboplatin	2	PhI	5	1	Palmoplantar erythema, pruritus	PhI	**17.4**	**32**	1	Fever, back pain
10	Docetaxel	2	CRR	1	0					0	—
11	Oxaliplatin	2	PhI	9	4	D1: pruritus, nasal congestion, nausea, abdominal pain	PhI	**5.8**	8	0	—
						D3: pruritus	PhI	NA	NA		
						D6: pruritus, nasal congestion, nausea	PhI	5.2	2		
						D8: pruritus	PhI	4.2	<2		
12	Rituximab	1	PhI	1	0	—				0	—

*IL-6, interleukin 6; D1, desensitization 1; D3, desensitization 3; etc.; NA, not available; PhI, phenotype I; CRR, cytokine release reactions; EPh, either phenotype.*

*Values in bold indicate significant elevations either above the reference limit or when the tryptase value during the reaction was greater than (BT × 1.2) + 2*.

Serum levels of tryptase and IL-6 were quantified in 5 out of 7 (71.4%) BTRs during desensitization. Two patients presented significant elevations of tryptase, including patient 11, allergic to oxaliplatin, who was identified by applying the formula (Basal Tryptase × 1.2 + 2); both elevations corresponded to PhI reactions. In addition to the increase in tryptase, a slight elevation of IL-6 was demonstrated during an immediate BTR; this patient had a non-immediate BTR suggestive of CRR several hours after finishing desensitization (Table 3).

## Discussion

The phenotype-based approach of precision medicine allows us to predict how patients will respond to treatment (7). We described the clinical characteristics of the CPh reactions and their evolution during the desensitization of several drugs that shared the appearance of an NIDHR but which, after successive exposures the phenotype, changed into IDHRs (6). Based on our observations, we make a series of recommendations on the allergological workup for this phenotype.

The characteristic change in the CPh makes it unique. Previous publications by Gómez et al. (2) and Torres et al. (3) describe accelerated reactions which appear 1-48 h after exposure to the drug, without associated changes in the phenotype and with evidence that the endotype involved in them is the activation of T lymphocytes. Our results suggest that initial CPh reactions always appear at least 6 h after exposure, fulfilling the criteria for an NIDHR (8). Thus, CPh is not a subtype of accelerated reactions, and the mechanisms that provoke them should be investigated.

In these patients, NIDHRs often developed on the first exposure to the drug and were characterized by the appearance of non-severe symptoms including maculopapular exanthema, erythema, and flushing. Several days after exposure, some patients also developed symptoms suggestive of cytokine release such as back pain, myalgias, arthralgias, chest tightness, and fever.

We showed that after developing an NIDHR, at least one subsequent exposure was needed before changing the phenotype to develop an IDHR. Most patients developed PhI reactions, especially those treated with platins (24, 25, 30), who presented the highest number of exposures, followed by MAb (27), supporting previous observations that indicate that IgE mediation is a fundamental mechanism in the development of DHRs to these drugs (9, 24, 27).

On the other hand, almost all taxane-treated patients developed CRR during the first exposures. Patients who converted from an NIDHR to PhI reaction had mostly presented with symptoms such as myalgia, arthralgia, malaise, and fever, while those who developed CRR had presented maculopapular exanthema, chest tightness or pain. These findings suggest that during NIDHRs, different cytokines on which the symptoms depend could be released. In the first group, the release of IFN-γ would predominate, while in the second group excessive amounts of other cytokines such as IL-6, TNF-α and IL-10 would be produced (31).

Regarding desensitization, the overall efficacy we observed, at 80.4%, was better than in previous publications, where it ranged from 74% (32) to 76.8% (27), which a priori suggests that this phenotype does not cause more BTRs. Our results support the idea that desensitization prevents the reappearance of serious, potentially life-threatening immediate reactions (33).

In addition, during BTRs we did not observe new changes in the phenotypes. Moreover, compared to Silver et al. (24), in our study patients who presented CRRs responded better to desensitization, perhaps due to the possible effect of desensitization on the components of the Src complex, present not only in mast cells but also in other cells of the innate immune system (34–36).

Regarding biomarkers, skin tests were very useful for identifying PhI reactions, even in patients who reacted after few exposures to the drugs. On the other hand, elevation of serum tryptase and IL-6 were related to the phenotypes involved in the reaction, supporting the idea that they are useful in the endophenotyping of the reactions (1, 11). In this sense, one patient who presented PhI BTR had a significant elevation of tryptase and a slight increase in IL-6, and hours later developed symptoms suggestive of cytokine release, which could be secondary to a delayed release of mediators by mast cells.

Previous observations about CPh suggest that the proper allergology workup after the NIDHRs using *in vivo* and *in vitro* tests (including skin test, patch tests and/or the quantification of biomarkers) could inform the decision on which patients would benefit from a challenge or desensitization (37) in order to avoid progression from a non-immediate to an immediate reaction (6). Although the information that can be gleaned from these tests is limited, the cytokine release enzyme linked to the ImmunoSpot (ELISpot) assay could be useful to identify the endotype in these reactions (38).

**Figure 1:**
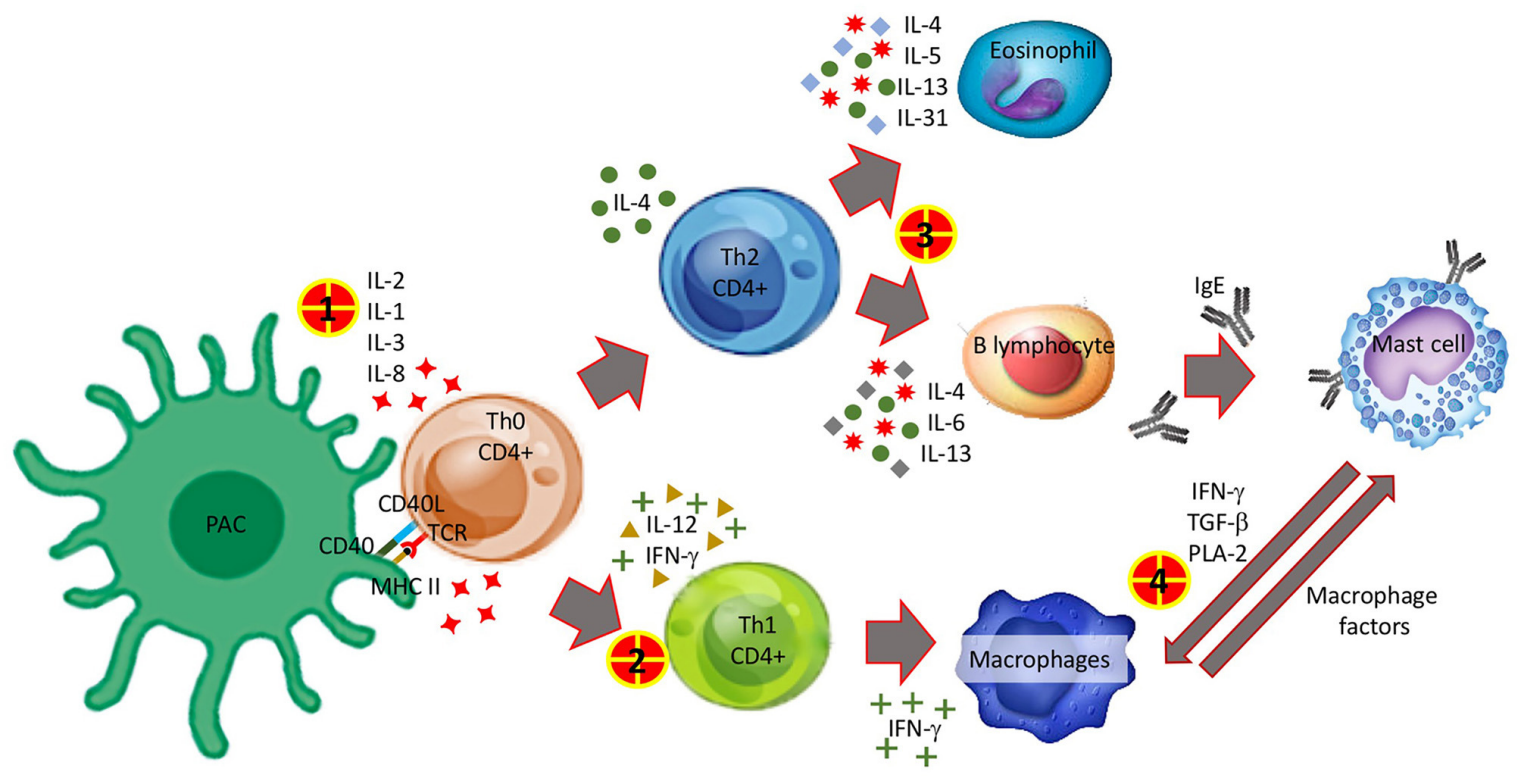
Possible mechanisms involved in the appearance of the converter phenotype. 1 Interleukins released after antigen presentation and lymphocyte differentiation to CD4+ Th1 and Th2 may be related to the occurrence of non-immediate reactions. 2 Th1 polarization. 3 Th2 polarization. 4 The pathway by which macrophages and monocytes are activated in cytokine release reactions is not entirely clear. On the one hand, it could be a non-predominant activation of Th1 that would allow these cells to discharge a large amount of cytokines responsible for the symptoms and, on the other hand, mast cells would establish a bridge with these and other cells of the innate immune system by releasing more cytokines that would amplify the immune response and contribute to symptoms.

Finally, although our objective was not to clarify the mechanisms by which CPh could develop, our observations allow us to hypothesize. A logical explanation involves both innate and acquired immunity. During the NIDHR, the cytokines released would be responsible for the symptoms and for the polarization of the reaction, triggering a Th1 response with the subsequent appearance of a CRR in successive exposures, or on the contrary the cytokines could produce a Th2 response, responsible for the activation of mast cells as an IgE-mediated response. There are connection pathways between mast cells and macrophages that could explain mixed phenotypes (Figure 1).

This descriptive study has several limitations. Firstly, our cohort is not representative of all patients with CPh because not all those who developed NIDHR were rechallenged, and some patients who finally changed their phenotype were not sent to evaluation by the allergist. Secondly, the size of the sample and the heterogeneity of the patients and drugs involved limit the performance of a robust statistical analysis and the extrapolation of the data. Thirdly, intravenous chemotherapeutic and biological agents are highly sensitizing drugs and the IDHR experienced by these patients could potentially be unrelated to the previous NIDHR. Finally, we did not use experimental methods that would allow us to verify our hypothesis, so we recommend carrying out basic studies that make it possible to elucidate the mechanisms that could be involved in this potential CPh as described in our study.

## Conclusions

Summarizing, CPh denotes a set of reactions characterized by an initial NIDHR and subsequent IDHRs after repeated exposures. These reactions, which had initially been described after exposure to taxanes, present a similar pattern after administration of other drugs, including platins and MAb.

The symptoms presented during the NIDHR may indicate the need for an early allergological evaluation to assess the risk of future IDHRs. Rapid drug desensitization could prevent conversion or, if this has already occurred, prevent the recurrence of serious or life-threatening immediate reactions.

The clinical characteristics of reactions, skin test results, and biomarkers can help predict responses to rapid drug desensitization, guiding clinicians on how to optimize therapy delivery while maintaining patient safety.

## Data Availability Statement

The original contributions presented in the study are included in the article/supplementary material, further inquiries can be directed to the corresponding author/s.

## Ethics Statement

The studies involving human participants were reviewed and approved by Ethics Committee for Research with Medicines of the Alicante Department of Health. The patients/participants provided their written informed consent to participate in this study.

## Author Contributions

TWJ-R, IL-C, RM-A, VS-G, PG-D, and JF-S contributed to conception and design of the study. FM, RM-A, and NM-B coordinated the data collection. FM analyzed the biomarkers results. RM-A, AB-SJ, SC-B, and NM-B organized the database and analyzed the results. TWJ-R wrote the first draft of the manuscript. All authors contributed to manuscript revision, read, and approved the submitted version.

## Conflict of Interest

The authors declare that the research was conducted in the absence of any commercial or financial relationships that could be construed as a potential conflict of interest.

## Publisher's Note

All claims expressed in this article are solely those of the authors and do not necessarily represent those of their affiliated organizations, or those of the publisher, the editors and the reviewers. Any product that may be evaluated in this article, or claim that may be made by its manufacturer, is not guaranteed or endorsed by the publisher.
